# Lipid Composition Drives Mutant Huntingtin Dimerization and Membrane Association: Insights from Computational Simulations

**DOI:** 10.3390/molecules31111965

**Published:** 2026-06-05

**Authors:** Catalin Nicoara, Emanuele Criscuolo, Angela De Cristofaro, Filomena Fezza, Mauro Maccarrone

**Affiliations:** 1 Department of Experimental Medicine, Tor Vergata University of Rome, Via Montpellier 1, 00121 Rome, Italy; catalin.nicoara@alumni.uniroma2.eu; 2 Institute for Complex Molecular Systems (ICMS), Department of Biomedical Engineering, Eindhoven University of Technology, 5600 MB Eindhoven, The Netherlands; e.criscuolo@tue.nl; 3 Department of Biology, University of Rome “Tor Vergata”, Via Della Ricerca Scientifica, 00133 Rome, Italy; angela.decristofaro@students.uniroma2.eu; 4 Department of Biotechnological and Applied Clinical Sciences, University of L’Aquila, Via Vetoio, 67100 L’Aquila, Italy; 5 European Center for Brain Research/Santa Lucia Foundation IRCCS, Via Del Fosso di Fiorano 64, 00143 Rome, Italy

**Keywords:** neurodegenerative diseases, mutant huntingtin exon 1, polyglutamine misfolding, molecular dynamics, cholesterol-dependent modulation, protein–membrane interactions, protein dimerization

## Abstract

Huntington’s disease (HD) is a neurodegenerative disorder caused by the expansion of the CAG trinucleotide in the exon 1 of the huntingtin gmodellerene. This abnormal expansion produces a mutant huntingtin (mHTT) protein with extended polyglutamine (polyQ) tracts. Although the molecular mechanisms underlying HD onset and progression remain poorly understood, aberrant folding, aggregation, and membrane interactions of mHTT are considered central to disease pathogenesis. In this study, we used molecular dynamics (MD) simulations to investigate the structural properties, dimerization propensity, and membrane lipid interaction of mHTT carrying 70 polyQ repeats (mHTT-Q70). Our analyses revealed that mHTT-Q70 retains partially structured α-helical conformations with increased flexibility within the polyQ domain, thus being predisposed to misfolding. Coarse-grained MD simulations further revealed a strong tendency of mHTT-Q70 to dimerize, indicating that early oligomerization may represent a critical step in protein aggregation. Interestingly, we show that membrane cholesterol content dose-dependently promotes dimeric mHTT-Q70—but not monomeric mHTT-Q70—association with neuronal membrane models, which was observed for 70% of simulation time at 40% cholesterol content. Such a cholesterol-dependent membrane binding of dimeric mHTT-Q70 suggests that membrane lipid composition may represent a critical checkpoint in the early stages of mHTT-Q70 aggregation, and of cytotoxicity thereof. Moreover, distinct neuronal membrane lipids like phosphatidylcholine, phosphatidylethanolamine, and phosphatidylserine differently contributed to mHTT-Q70 binding, highlighting the complexity of such a lipid-dependent modulation. Taken together, these findings underscore the dynamic interplay between polyQ-driven misfolding, dimerization, and membrane lipids in HD pathogenesis, suggesting that modulation of membrane composition, and in particular of cholesterol levels, may be a novel action point to design therapeutic drugs for HD.

## 1. Introduction

Huntingtin (HTT) is a large soluble protein found in humans and other mammals [1,2]. Human HTT is particularly abundant in the brain, where it interacts with different effector proteins in order to mediate many neuronal functions [3,4]. Although its precise function is not yet fully understood, HTT appears to be involved in a wide variety of biological processes, from early embryonic development and intracellular vesicular trafficking to regulation of gene transcription and neurotransmission [1,2,3,4,5].

Human HTT comprises 3144 amino acids and holds a polyglutamine (polyQ) domain within its N-terminal domain, which usually contains 11–36 glutamine residues beginning at the 18th amino acid position [4,6,7,8]. The polyQ tract is encoded by CAG trinucleotide repeats in the exon 1 of the huntingtin gene on chromosome 4p16.3. Expansion of the polyQ length beyond 36 glutamine repeats (>36Q) generates a mutant huntingtin (mHTT), which leads to the autosomal dominant Huntington’s disease (HD) [8].

HD is a neurodegenerative disorder characterized by progressive motor dysfunction, cognitive decline, and psychiatric symptoms [8,9,10,11]. It is caused by the insoluble nature of expanded polyQ residues, which render mHTT prone to aggregation and cause the formation of toxic assemblies. Protein aggregation disrupts cellular processes that ultimately lead to neuronal death, particularly in the striatum and cortex of the brain [12,13,14,15]. Furthermore, an inverse correlation between age of onset and severity of the disorder has been reported, with larger CAG expansions promoting earlier onset and more severe symptoms, thus resulting in brain neurodegeneration [16].

As yet, the molecular mechanisms underlying HD onset and progression have not been fully elucidated, though the most widely accepted hypothesis is that polyQ expansion in exon 1 leads to atypical folding behaviors, thus interfering with normal neuronal functions or cell survival [17]. It should be noted that HD pathogenesis is multifactorial, and that aggregation and membrane interactions represent only two of several proposed pathogenic mechanisms. Additional contributing factors include transcriptional dysregulation, mitochondrial dysfunction, oxidative stress, impaired axonal transport, and excitotoxicity [18,19,20,21], all of which may act in concert with protein misfolding to drive neuronal death. Recent studies indicate that these misfolded aggregates sequester essential cellular components, including transcription factors and chaperone proteins, thereby exacerbating cell dysfunction and contributing to toxicity and neuronal death [22]. Both wild-type and mutant HTT are ubiquitously expressed across tissues [23,24,25,26]. In neurons, HTT is mainly localized in the cytoplasm, where it can be found in both soluble and membrane-associated forms. Notably, structural analysis predicts that HTT is associated with membranes through its N-terminus [27,28,29]. Furthermore, the multiple subcellular localizations and the presence of membranous structures within the aggregates strongly suggest a direct interaction of HTT with lipid bilayers [30,31]. In line with this, increasing evidence indicates that membrane-HTT interactions may be crucial in the early stages of HD, because cholesterol-enriched microdomains like lipid rafts—and more generally membrane composition—appear to modulate the dynamics of HTT aggregation [32,33,34,35,36].

Interestingly, the accumulation of proteins into high molecular weight fibrillar assemblies is a hallmark of several neurodegenerative disorders such as Alzheimer’s and Parkinson’s diseases, as well as HD [12,13,14,15,27,37]. During the last decade, it has become evident that these fibrillar aggregates can propagate between cells in a prion-like manner, triggering misfolding and aggregation of endogenous proteins in the recipient cells [38]. In this context, membrane lipid composition has emerged as a critical determinant of amyloid-lipid interactions, as well as of promotion of fibrillogenesis of β-amyloid and α-synuclein, respectively, the main aggregating proteins in Alzheimer’s and Parkinson’s diseases [39,40,41,42,43,44,45]. Taken together, these findings, although derived from other amyloidogenic proteins, raise the possibility that mHTT aggregation may be similarly influenced by the lipid environment, potentially leading to neuronal toxicity and hence affecting disease onset and progression [35,46,47]. Cholesterol, an essential structural component of biological membranes, seems particularly relevant for the regulation of membrane fluidity, trafficking, and signaling [48,49,50,51]. Remarkably, abnormalities in cholesterol metabolism and homeostasis have been repeatedly observed in cellular and animal models of HD [46,47,52,53], as well as in tissues isolated from HD patients [54,55,56]. However, to date, the impact of changes in cholesterol content associated with HD remains a matter of debate [57,58,59]. While many studies have documented a reduced cholesterol biosynthesis and content in HD models [55,58,60,61], others have independently reported an increase or accumulation of cholesterol in specific subcellular compartments of HD models [55,62]. This apparent discrepancy highlights the need to better address the role of lipid homeostasis in HD, and to ascertain whether membrane lipids—and in particular cholesterol—may regulate key players of HD such as mHTT.

To address these questions, computational approaches like molecular dynamics (MD) simulations have emerged as powerful and internationally validated tools for investigating protein–membrane interactions and aggregation mechanisms at atomic resolution [63,64]. MD simulations enable the detailed characterization of conformational dynamics, lipid-protein interactions, and the effects of membrane composition on protein behavior, providing mechanistic insights that are often difficult to obtain experimentally [65,66,67]. Notably, several landmark studies on amyloid proteins and membrane-associated processes have established the scientific rigor and predictive value of purely in silico investigations, demonstrating that computational findings can drive our understanding of complex biological phenomena even in the absence of direct experimental validation [63,64,65,66]. In this context, MD simulations represent an ideal approach to systematically explore how cholesterol modulates mHTT behavior at the molecular level. The present investigation aimed to fill this knowledge gap. In this context, a polyQ length of 70 glutamine residues was selected, as it represents a clearly pathogenic expansion while avoiding extremely long polyQ tracts associated with rapid aggregation and overwhelming neurotoxicity [30,68,69,70,71].

## 2. Results

### 2.1. Structural Characterization and Analysis of Dimerization Propensity of mHTT

Molecular dynamics (MD) simulations were performed to investigate the structural dynamics and folding behavior of wild-type and mHTT proteins, focusing on exon 1, the region that harbors CAG trinucleotide expansion. The polyglutamine (polyQ) tract was set to 70 glutamine residues (Q70) to model a pathogenic expansion [30,72,73]. As a starting model, we used the HTT exon 1 structure deposited in the Protein Data Bank (PDB) under the code 4FEB, which contains the first 17 N-terminal residues (N17), followed by a polyQ tract of 36 glutamine residues. To model the pathogenic mHTT variant, the polyQ tract was computationally extended from 36 to 70 repeats (mHTT-Q70) by using the Modeller 9.23 software, while preserving the native N17 structure. The resulting full-length exon 1 model (residues 1–87, i.e., N17 + Q70) was then subjected to MD simulations to investigate its conformational dynamics and membrane interactions.

**Figure 1:**
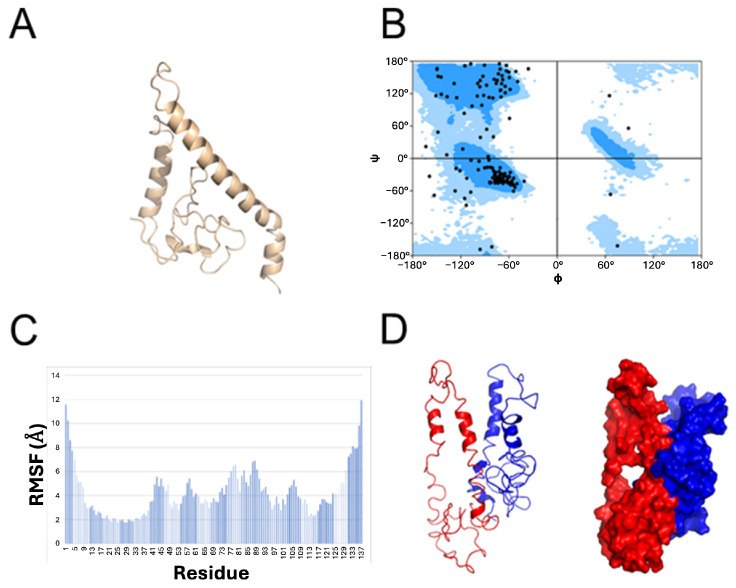
(**A**) Structural modeling and validation of mHTT-Q70. Modeled mHTT-Q70, based on PDB 4FEB (36Q) and extended via Modeller, after 100 ns of standard MD and 250 ns of accelerated MD, showing multiple α-helices connected by loops. (**B**) Ramachandran plot depicting the distribution of φ and ψ angles for all residues. (**C**) Residue flexibility of mHTT-Q70. Per-residue RMSF values were calculated from the MD trajectory, illustrating the flexibility of each residue along the mHTT polypeptide chain. (**D**) CG-MD simulation of mHTT dimerization: (left) backbone trace representation of two mHTT-Q70 molecules (in red and blue, respectively), illustrating overall protein–protein contacts; (right) space-filled model of two mHTT-Q70 molecules (in red and blue, respectively), illustrating overall protein–protein contacts.

The mHTT-Q70 showed a stable structure with multiple α-helices interconnected by loops, as illustrated in Figure 1A. Despite the elongated polyQ tract, these secondary structures suggest partially structured conformations characterized by α-helical segments, consistent with the known dynamic and partially disordered nature of exon 1 HTT. This observation is consistent with previous reports on intermediate aggregation states [74,75]. Structural validation via the Ramachandran plot confirmed that it was a high-quality model, with only 0.06% of residues falling in disallowed regions (Figure 1B). Additionally, flexibility analysis through Root Mean Square Fluctuation (RMSF) plots revealed increased fluctuations in residues 41–47, 55–62, 71–95, and 103–108, particularly within the polyQ region (Figure 1C). Such an increased flexibility aligns with the known intrinsic disorder of expanded polyQ domains, which contributes to misfolding and aggregation [74,75].

Next, Coarse-Grained Molecular Dynamics (CG-MD) simulations of two mHTT-Q70 proteins in an aqueous solution for 5 µs revealed a strong tendency to aggregation, with homodimer formation occurring progressively throughout the simulation (Figure 1D). In particular, extensive protein–protein contacts were observed, accompanied by conformational deviations from the initial folded state (Figure 1D). Such a structural rearrangement underscores the dynamic nature of mHTT-Q70 dimerization and its potential role in aggregation.

Taken together, these findings reinforce the hypothesis that polyQ expansion promotes intermolecular interactions, which is widely recognized as a key factor in HD pathology [30,76,77,78].

Given the strong dimerization propensity observed in aqueous solution (Figure 1D), we next investigated whether the oligomerization state may influence mHTT-Q70 interaction with membranes. Previous studies have suggested that membrane binding may promote mHTT aggregation, but the specific role of membrane lipids—and in particular of cholesterol—in modulating this process remained unexplored. Therefore, we conducted systematic CG-MD simulations to examine how both monomeric and dimeric mHTT interact with neuronal membrane models of physiologically relevant lipid compositions.

### 2.2. Role of Cholesterol on mHTT-Q70–Neuronal Membrane Interaction

Several studies have reported that mHTT interacts with the cell membrane [79,80,81,82,83,84,85]. Notably, it has been demonstrated that mHTT preferentially binds to specific lipid species such as phosphatidylinositol(3,4,5)-trisphosphate (PIP_3_), with binding affinity increasing alongside polyQ tract length [27]. It has been reported that membrane interaction is mediated via the N-terminal α-helix of mHTT, providing a potential therapeutic target for HD [35]. Furthermore, the addition of cholesterol to synthetic lipid vesicles was shown to modulate mHTT interaction with the membrane, either increasing or decreasing binding depending on membrane composition [46].

Against this background, we investigated the interaction of both monomeric and dimeric mHTT-Q70 with neuronal membrane models, whose lipid composition was chosen according to Ingòlfsson and colleagues, who showed that cholesterol is the most abundant lipid (41%) in both inner and outer leaflets [86,87,88,89]. To assess whether cholesterol content may impact on mHTT-Q70-membrane interaction, 1 µs CG-MD simulations were performed at increasing (0%, 20%, 40%) cholesterol concentrations.

**Figure 2:**
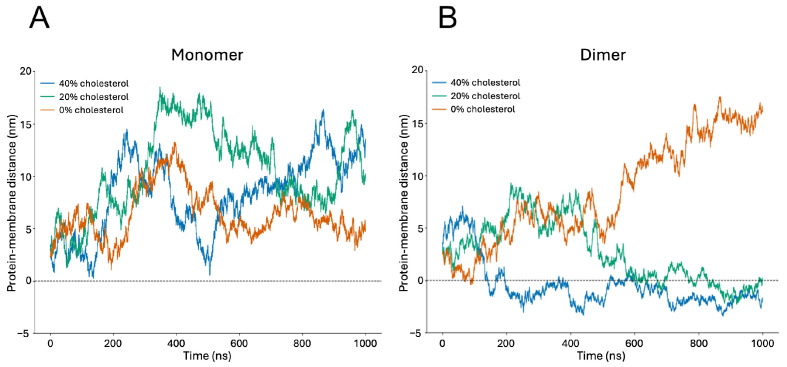
Protein–membrane distance of mHTT-Q70 monomers and dimers at varying cholesterol concentrations. Time-course evolution of the distance between monomeric (**A**) and dimeric (**B**) mHTT-Q70 and the membrane during 1 µs Coarse-grained MD simulations at different cholesterol concentrations (0%, 20%, 40%). Protein–membrane distance is expressed in nanometers (nm) and is shown as a function of simulation time.

Monomeric mHTT-Q70 failed to interact with the neuronal membrane models at any cholesterol concentration (Figure 2A), whereas dimeric mHTT-Q70 showed a strong cholesterol-dependent interaction. Indeed, in the absence of cholesterol, no membrane interaction of dimeric mHTT-Q70 was observed throughout the simulation. At 20% cholesterol, the contact of the mHTT-Q70 dimer with the membrane was observed in the later stages of the simulation, and at 40% cholesterol, the mHTT-Q70 dimer remained bound to the membrane for 70% of the simulation time (Figure 2B).

**Figure 3:**
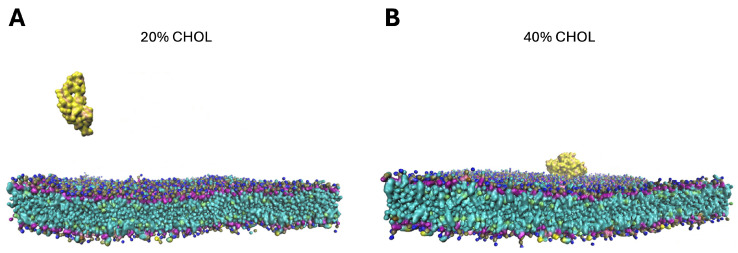
Comparison of dimer (yellow)–membrane interactions at different cholesterol concentrations during the early stages of the simulation at 20% cholesterol (**A**) and 40% cholesterol (**B**).

In particular, representative snapshots from the early stages of the simulation show that at 20% cholesterol (Figure 3A), the dimer remains detached from the membrane, indicating weak or no stable interaction with the membrane surface. In contrast, at 40% cholesterol (Figure 3B), the dimer is bound to the membrane, suggesting that higher cholesterol content promotes stable association of the dimer with the lipid bilayer.

These findings demonstrate a cholesterol-dependent mechanism of mHTT-Q70 binding to the membranes, where protein dimerization plays a crucial role. They also provide further insights into the molecular determinants of mHTT aggregation, which is known to impact HD pathogenesis [4,90,91,92,93].

### 2.3. Effect of Distinct Lipids on the Interaction of mHTT-Q70 with Neuronal Membranes

**Figure 4:**
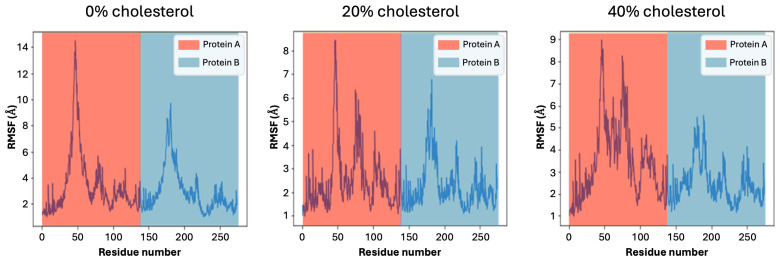
RMSF plots of protein A and protein B simulated in membranes containing 0%, 20%, and 40% cholesterol. At 40% cholesterol, protein B exhibits markedly reduced flexibility (lower RMSF values) compared to protein A, consistent with direct membrane binding. Protein A, which remains in solution without membrane contact, maintains high flexibility at all cholesterol concentrations. The stabilization of protein B correlates with its sustained membrane association (shown in Figure 2B), thus demonstrating that membrane binding stiffens the protein structure.

To further explore the interaction between dimeric mHTT-Q70 and the membrane surface, we first analyzed how each monomer (protein A and protein B) within the dimer interacts with the membrane at different cholesterol concentrations (Figure 4). The residue-level flexibility was quantified by RMSF, which showed overt differences between protein A and protein B at increasing (0%, 20%, and 40%) cholesterol concentrations.

Protein A appeared more flexible, consistent with a lack of membrane interaction, whereas protein B appeared more stable in the presence of 40% cholesterol, highlighting a cholesterol-dependent effect on protein dynamics (Figure 4).

**Figure 5:**
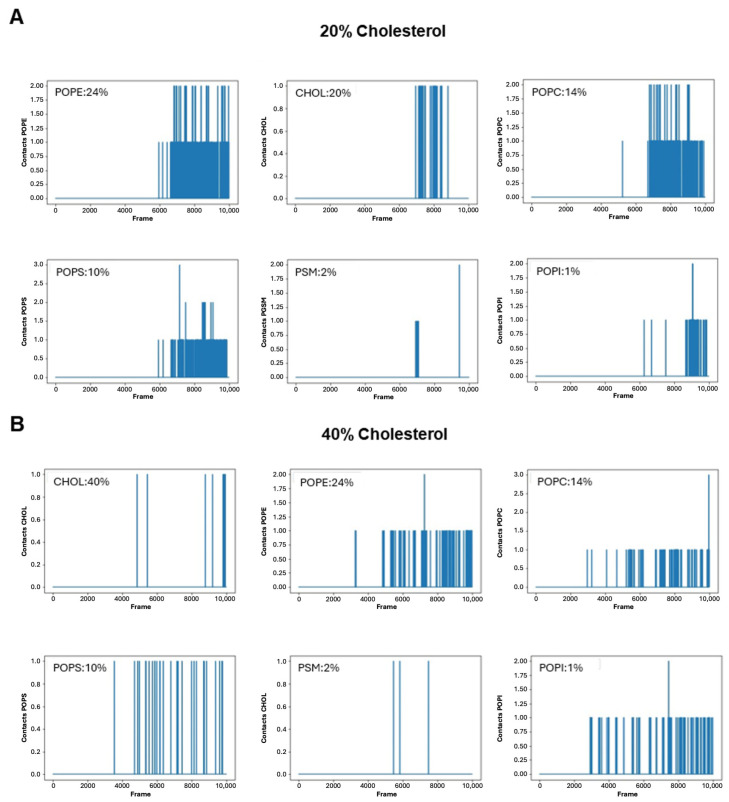
Lipid-specific contacts of mHTT-Q70. Time-resolved number of contacts between mHTT-Q70 proteins and individual lipid species: Cholesterol (CHOL), POPC, POPS, PSM, and POPI at 20% (**A**) and 40% (**B**) of CHOL. Contacts were calculated per simulation frame, illustrating the contribution of each lipid type to protein–membrane interactions at different cholesterol content.

Then, different lipid compositions were used to interrogate the influence of lipid diversity on protein–membrane interactions. In addition to cholesterol, major components of both leaflets of neuronal membranes are known to include phosphatidylcholine (POPC, 1-palmitoyl-2-oleoyl-*sn*-glycerol-3-phosphocholine), phosphatidylethanolamine (POPE, 1-palmitoyl-2-oleoyl-*sn*-glycerol-3-phosphoethanolamine), and palmitoylsphingomyelin (PSM, *N*-palmitoyl-sphingosine-1-phosphocholine), while phosphatidylserine (POPS, 1-palmitoyl-2-oleoyl-*sn*-glycerol-3-phospho-L-serine), phosphatidylinositol (POPI, 1-palmitoyl-2-oleoyl-*sn*-glycerol-3-phosphoinositol), and glycolipids (OPSG) are asymmetrically distributed between leaflets, with glycolipids confined exclusively to the outer leaflet [86,94,95]. On this basis, specific lipid contacts were analyzed to ascertain their contribution to protein–membrane interaction. Time-resolved profiles revealed distinct binding patterns for POPE, cholesterol, POPC, POPS, PSM, and POPI at different cholesterol concentrations, demonstrating that indeed the composition of the lipid bilayer influences the interaction of mHTT-Q70 with the membrane (Figure 4). More frequent signals were observed for lipids that are more abundant in the membrane (such as POPE, POPC, and POPS) [86], while less represented species like PSM and POPI [86] showed only occasional interactions. Glycolipids, present exclusively in the outer leaflet, did not establish any detectable interaction with mHTT-Q70 across any of the simulated systems. Finally, cholesterol helped to stabilize the dimer at the membrane surface, especially at higher concentrations (Figure 5A,B).

Interestingly, the differential flexibility observed between the two monomers within the dimer provides direct evidence for asymmetric membrane binding. At 40% cholesterol, protein B displayed reduced RMSF values compared to protein A, indicating that protein B makes stable contact with the lipid bilayer while protein A remains predominantly in the aqueous phase (Figure 4).

Overall, our comprehensive computational approach provides valuable insights into the structural dynamics and interactions of the mutant HTT protein, shedding light on their potential role in HD onset and progression.

## 3. Discussion

Our MD simulations provide valuable insights into the structural behavior and aggregation propensity of the 70 polyQ-repeated mHTT. These simulations demonstrate that the mutant protein preserves a partially structured conformation, characterized by multiple α-helices connected by flexible loops. Such a structural organization of the final stable conformation (Figure 1A) is further supported by the Ramachandran distribution of backbone φ and ψ angles (Figure 1B). Notably, an increased flexibility is observed in mHTT-Q70 (Figure 1C), supporting the idea that the expanded polyQ domain introduces a local disorder. Protein flexibility is closely related to misfolding and aggregation [96,97,98,99], providing a likely structural basis for the pathogenicity of polyQ expansions. In this context, a pronounced aggregation propensity of two mHTT-Q70 molecules emerged over 5 µs of CG-MD simulations in aqueous solution, performed to investigate the dimerization potential of mHTT-Q70 (Figure 1D). This structural reorganization is consistent with the hypothesis that dimerization may represent an early step in the aggregation process, although whether this translates to higher-order oligomer formation in a cellular context remains to be experimentally verified. Furthermore, significant structural deviations from the initial monomeric conformation were observed upon dimerization, highlighting the dynamic nature of protein–protein interactions observed in these simulations, with potential implications for HD pathogenesis [100,101].

To date, several studies have reported the ability of mHTT to interact with cell membranes [79,80,81,82,83,84,85]. For instance, it has been demonstrated that mHTT binds to the lipid bilayer with a marked selectivity towards distinct lipid species such as PIP_2_; this interaction is further strengthened when increasing polyQ length in mHTT [27,102,103]. While some physiological functions attributed to huntingtin, including intracellular trafficking and transcriptional regulation, are primarily associated with the wild-type protein, accumulating evidence indicates that the membrane-binding properties of mHTT are retained and even exacerbated upon polyQ expansion, contributing to pathological outcomes [4,5,104,105]. Available evidence suggests that the N-terminal α-helix of mHTT mediates membrane binding, thus providing insights into specific structural elements that drive protein–membrane interactions and that could become potential therapeutic targets beyond mHTT [4,5,105].

Together, these studies underscore the importance of lipid composition in modulating protein–membrane interaction and suggest a link between membrane lipids and HD. Consistently, our analysis of protein–membrane dynamics revealed that monomeric mHTT-Q70 is unable to interact with neuronal membranes at different cholesterol concentrations, whereas the mHTT-Q70 dimer clearly binds them in a cholesterol-dependent manner (Figure 2). Cholesterol is known to reduce membrane fluidity [106,107] by locally modulating membrane deformation and binding affinity of proteins [108]. In the context of mHTT-Q70, cholesterol-induced membrane condensation may increase bilayer surface rigidity, thereby providing a more stable docking platform for the amphipathic N17 helix. The dimeric form, presenting a larger and geometrically more complementary membrane-interacting surface than the monomer, may be better positioned to exploit this effect cooperatively, while the monomeric form, inherently more flexible, may be unable to overcome the entropic cost of stable membrane association. In keeping with this knowledge, residue flexibility analysis of mHTT-Q70 (Figure 4) indicates that the dimer is more stable at 40% cholesterol—that is, its physiological concentration [86,95,109,110]—whereas the monomer remains highly flexible and unable to anchor to the membrane. Furthermore, lipid-specific contacts of mHTT-Q70 show differential contributions of POPC, POPE, POPS, PSM, POPI, and cholesterol itself (Figure 5), suggesting that lipid asymmetry and electrostatics may drive selective interactions. In this regard, it is noteworthy that POPS and POPI, both carrying a net charge of −1e per molecule at physiological pH and confined exclusively to the inner leaflet, may electrostatically attract the positively charged N-terminus of mHTT-Q70, whose N17 amphipathic helix has been established as the primary membrane-anchoring domain [35].

Importantly, the differential behavior of the two mHTT monomers within the membrane-bound dimer points to an asymmetric binding mode. One monomer appears to act as a membrane anchor, displaying reduced flexibility and stable lipid contacts, while the second monomer remains predominantly exposed to the aqueous phase. This asymmetric, “partially inserted” mode may represent an intermediate state in which one monomer acts as a membrane anchor while the other remains solvent-exposed. Speculatively, such a configuration could facilitate the recruitment of additional mHTT-Q70 molecules, although this hypothesis extends beyond what the present simulations directly demonstrate. It should be noted, however, that this asymmetric behavior was observed in a single trajectory per system, and cannot be formally verified for reproducibility in the absence of independent replicas. The observed asymmetry may therefore partly reflect stochastic effects related to the initial orientation of the two monomers relative to the membrane surface or limited conformational sampling within the 1 µs timescale. While the persistence of differential RMSF values throughout the trajectory at 40% cholesterol suggests that this is not a transient fluctuation, alternative explanations, including stochastic docking geometry and sampling bias, cannot be excluded. Future studies employing multiple independent replicas and enhanced sampling methods will be necessary to determine whether this asymmetric binding mode is a robust, reproducible feature of mHTT-Q70 dimer–membrane interaction.

Overall, these observations highlight that membrane composition may influence both the extent of mHTT-Q70 binding and the stabilization of oligomeric protein intermediates on the membrane surface, which may influence the aggregation behavior of mHTT at membrane surfaces, with potential but as yet uncharacterized implications for cellular toxicity.

Several limitations of the present study should be acknowledged. First, the use of the MARTINI 3 coarse-grained force field, while enabling microsecond-scale simulations of complex membrane systems, sacrifices atomic-level detail. Specific hydrogen bonding patterns, side-chain chemistry, and short-range electrostatic interactions are not fully resolved, which is particularly relevant given the proposed role of electrostatics in driving selective lipid-protein contacts. Furthermore, while widely used for membrane simulations, the MARTINI 3 coarse-grained force field is known to overestimate protein aggregation propensity in some contexts [111,112]. The observed dimerization behavior should therefore be interpreted with this caveat in mind, and future studies using orthogonal force fields or all-atom simulations would help validate these findings.

Second, the simulation timescale of 1 μs for membrane systems, although appropriate for observing cholesterol-dependent association events, may be insufficient to capture rare binding events. Furthermore, each simulation system was represented by a single production trajectory without independent replicas, which limits the statistical assessment of the observed membrane-binding behavior. Future studies should include multiple independent runs to confirm the reproducibility of the reported findings.

Additionally, the all-atom MD simulations were limited to 100 ns plus 250 ns of accelerated MD, which may be insufficient to fully characterize the conformational landscape of an intrinsically disordered polyQ system.

In particular, it cannot be excluded that monomeric mHTT-Q70 would associate with the membrane on longer timescales. Third, only a single pathogenic polyQ length (Q70) was investigated; the cholesterol-dependent membrane interaction described here may differ quantitatively or qualitatively for intermediate polyQ expansions, such as 40Q or 55Q. Fourth, a complete mechanistic characterization of the cholesterol effect, including membrane order parameters, free energy profiles, and radial distribution functions, was not performed and remains an important objective for future computational work. Finally, the present study is entirely computational, and experimental validation through in vitro and in vivo approaches will be necessary to confirm the relevance of these findings in biologically and physiologically relevant settings.

Future in vitro and in vivo studies—currently precluded by the lack of purified mHTT used in this investigation—will be necessary to validate the present in silico results, and to elucidate the contribution of additional membrane lipids to mHTT modulation. Moreover, future studies should assess potential lipid-targeted interventions able to mitigate mHTT-dependent cytotoxicity and neuronal dysfunction, with an obvious impact on HD onset and progression. On a final note, it should be stressed that most of the membrane lipids used in this study are rather common components of plasma membranes, raising the possibility that the present findings may extend to non-neuronal cellular contexts, a conjecture that requires experimental testing.

## 4. Conclusions

The present study provides novel insights into the molecular mechanisms underlying the aggregation propensity and membrane interaction of mHTT. Through MD simulations, we observed that the expansion of the polyQ tract (with Q70 expansions) significantly influences the structural stability of the protein, leading to increased flexibility in critical regions. Such a structural disorder appears to predispose mHTT-Q70 to misfolding and aggregation, with α-helices and flexible loops coexisting in a partially structured conformation. Additionally, CG-MD simulations revealed a strong tendency of mHTT-Q70 to form dimers, further highlighting its intrinsic aggregation potential. In particular, dimerization was found to induce significant conformational changes compared to the monomer, suggesting that early oligomerization of mHTT-Q70 may represent a step along the aggregation pathway, a hypothesis that warrants future experimental validation.

The role of lipid diversity in modulating mHTT-Q70 interaction with membranes seems a key outcome of our study, demonstrating that specific lipids—particularly cholesterol—can influence mHTT-Q70 binding to neuronal membranes. In particular, changes in cholesterol content (0%, 20%, 40%) revealed that monomeric mHTT-Q70 does not interact with the lipid bilayer, whereas dimeric mHTT-Q70 does so in a cholesterol-dependent manner, with sustained interactions occurring at higher cholesterol concentrations.

Overall, mHTT-Q70 showed typical structural features and stability, laying the groundwork for further investigations into its functional implications in HD pathogenesis. Our findings underscore the intricate relationship between protein structure, aggregation, and membrane lipid composition in HD onset and progression.

Of note, the interplay between membrane composition and protein aggregation observed for mHTT-Q70 may extend beyond HD to other neurodegenerative proteinopathies. Indeed, the propensity to form fibrillar aggregates at membrane interfaces has been documented for several amyloidogenic proteins, including α-synuclein in Parkinson’s disease [113,114], amyloid-β in Alzheimer’s disease [115,116], tau protein [117,118], and the prion protein [119,120]. Furthermore, cholesterol-mediated modulation of protein–membrane interactions is not limited to aggregation-prone proteins, as it has been observed also for other membrane-binding proteins like cyclooxygenases [121], lipoxygenase [122,123], and fatty acid amide hydrolase [124,125]. The observed cholesterol-dependent mHTT-Q70 binding to membranes may offer a potential point of action for future therapeutic investigations. More generally, the present results extend an ever-growing body of evidence that links structural instability, aggregation, and cytotoxicity in polyQ-expanded proteins [28,34,35,36,126], paving the way for future experimental validations and drug discovery efforts.

## 5. Methods

### 5.1. Protein Modeling

The initial structure of huntingtin (HTT) exon 1 was obtained from the PDB (PDB ID: 4FEB), which includes the 17-residue N-terminal helix (N17) followed by a 36-residue polyglutamine (polyQ) tract. To generate the pathological variant, the polyQ tract was extended from 36 to 70 glutamine residues using MODELLER 9.23 (University of California, San Francisco, CA, USA). The unmodified 4FEB structure (N17 + 36Q) served as the wild-type HTT (wHTT) reference for all-atom simulations. Both structures were processed with the CHARMM-GUI web server (MacKerell laboratory, University of Maryland, College Park, MD, USA) [127] for system assembly.

### 5.2. All-Atom Molecular Dynamics Simulations

All-atom MD simulations were performed with GROMACS 2021.4 (BIOSON Research Institute and Laboratory of Biophysical Chemistry, University of Groningen, Groningen, The Netherlands) [128] using the CHARMM36m force field [129]. The protein (HTT and mHTT-Q70) was solvated in a cubic simulation box with explicit TIP3P water molecules. Na^+^ and Cl^−^ ions were added to neutralize the system and achieve a physiological ionic concentration of 0.15 M NaCl.

### 5.3. Energy Minimization

The system was energy-minimized using the steepest descent algorithm for a maximum of 5000 steps, with harmonic position restraints applied to backbone atoms (force constant 400 kJ mol^−1^ nm^−2^) and side-chain atoms (40 kJ mol^−1^ nm^−2^). Long-range electrostatics were treated with the Particle Mesh Ewald (PME) method (r_coulomb = 1.2 nm); van der Waals interactions were evaluated with a Force-switch scheme (r_vdw,switch = 1.0 nm, r_vdw = 1.2 nm).

### 5.4. Equilibration

Following energy minimization, the system was equilibrated under NVT conditions for 500 ps (500,000 steps, dt = 1 fs) at 300 K using the Nosé–Hoover thermostat (τ_t = 1.0 ps). Position restraints were maintained on backbone (400 kJ mol^−1^ nm^−2^) and side-chain (40 kJ mol^−1^ nm^−2^) atoms throughout equilibration to allow solvent relaxation. Initial velocities were generated from a Maxwell–Boltzmann distribution at 300 K. The same electrostatic and van der Waals parameters were used during equilibration and production.

### 5.5. Production Runs

Production runs were performed in the NVT ensemble for 100 ns (50,000,000 steps; dt = 2 fs). To enhance conformational sampling of the extended polyQ domain, an additional 250 ns of accelerated MD [130] was performed.

### 5.6. Accelerated Molecular Dynamics Simulations of mHTT-Q70

To further enhance conformational sampling of the expanded polyglutamine domain, accelerated molecular dynamics (aMD) simulations were performed on the mHTT-Q70 construct. The equilibrated structure obtained from the classical MD simulations described above was used as the starting configuration. Accelerated simulations were carried out using NAMD version 2.14, while maintaining the same force field and system configuration described for the classical MD simulations. Prior to applying the acceleration potential, a 50 ns conventional MD simulation was performed to estimate the energetic parameters required for aMD. In the aMD framework, the original potential energy surface *V*(*r*) is modified by adding a boost potential ∆*V*(*r*) whenever the system potential falls below a predefined energy threshold *E*. In this work, a dihedral boost scheme was applied, in which the acceleration potential acts on the torsional component of the potential energy to enhance sampling of backbone and side-chain conformations. The boost potential is defined as:

$$∆V\left(r\right)=\;\frac{{\left(E-V(r)\right)}^{2}}{a+(E-V\left(r\right))}$$

where *E* represents the reference energy level and α controls the magnitude and smoothness of the applied boost potential. The parameters *E* and α were determined from the average dihedral potential energy calculated from the preceding 50 ns classical MD trajectory. Using these parameters, accelerated MD simulations were performed for 250 ns to promote exploration of conformational states that may be difficult to access through conventional MD simulations. All remaining simulation parameters were kept consistent with those used in the classical MD simulations.

### 5.7. Coarse-Grained Molecular Dynamics Simulations

Coarse-grained MD (CG-MD) simulations were performed with GROMACS 2021.4 using the MARTINI 3 coarse-grained force field [131]. All CG systems were assembled using the CHARMM-GUI web server [127]. Electrostatic interactions were treated with a reaction-field scheme (ε_r = 15, r_coulomb = 1.1 nm) and van der Waals interactions with a Potential-shift-Verlet cutoff modifier (r_vdw = 1.1 nm), consistent with MARTINI 3 parameterization.

### 5.8. Dimerization Simulations in Aqueous Solution

To characterize the dimerization propensity of mHTT-Q70, two coarse-grained mHTT-Q70 molecules were placed in an aqueous simulation box and subjected to a 5 µs CG-MD production run. Temperature was maintained at 303.15 K using the velocity-rescaling thermostat [132] with τ_t = 1.0 ps applied to protein and solvent groups. Protein–protein contact analysis was performed over the full trajectory to characterize the kinetics and structural features of homodimerization.

### 5.9. Membrane System Preparation

Neuronal membrane models were constructed based on the lipid composition reported by Ingólfsson and colleagues [86], preserving the asymmetric distribution across leaflets: the outer leaflet comprised cholesterol (CHOL, 41%), POPC (1-palmitoyl-2-oleoyl-sn-glycero-3-phosphocholine, 24%), POPE (1-palmitoyl-2-oleoyl-*sn*-glycero-3-phospho ethanolamine, 12%), OPSG (glycolipids, 12%), and PSM (palmitoylsphingomyelin, 9%), while the inner leaflet comprised CHOL (41%), POPE (24%), POPC (14%), POPS (10%), PSM (2%), and POPI (1%). To systematically assess the effect of cholesterol on mHTT-Q70–membrane interactions, three compositions were examined with cholesterol mole fractions of 0%, 20%, and 40%, with the relative proportions of the remaining lipid species adjusted accordingly. The three cholesterol mole fractions were selected to represent distinct physiological and pathological contexts: 0% cholesterol serves as a negative control to isolate the contribution of cholesterol to protein–membrane interactions; 40% corresponds to the physiological cholesterol content of neuronal plasma membranes [86,95,109,110]; and 20% represents an intermediate concentration to assess the dose-dependence of the cholesterol effect.

Protonation states were assigned using CHARMM-GUI default settings at physiological pH 7.4. Protein termini were left uncapped, resulting in a charged N-terminus (+1e) and C-terminus (−1e). Among the lipid species, POPS and POPI carry a net charge of −1e per molecule in the MARTINI 3 parametrization, while all remaining lipids (POPC, POPE, PSM, CHOL, and glycolipids) are electrically neutral. The total system charge was neutralized by Na^+^ and Cl^−^ counter-ions, with an ionic strength of 0.15 M NaCl.

Both monomeric and dimeric mHTT-Q70 were incorporated in each membrane model, yielding six independent simulation systems. For the dimeric systems, two isolated mHTT-Q70 monomers were placed independently in the aqueous phase in proximity to the membrane surface. Dimerization was not imposed as an initial condition but was allowed to occur spontaneously during the simulation, enabling assessment of both dimerization propensity and membrane association in a single trajectory.

### 5.10. Energy Minimization of Membrane Systems

Each membrane system underwent a three-stage energy minimization protocol. The first stage (5000 steps of steepest descent) to remove severe steric clashes; Van der Waals and electrostatic cutoffs were set to 1.5 nm in this stage. The second stage (5000 steps) repeated the steepest descent without soft-core interactions, switching to standard MARTINI cutoffs (r_coulomb = r_vdw = 1.1 nm). The third stage (50,000 steps) was a final standard steepest descent at the same cutoffs to ensure full convergence of the membrane system.

### 5.11. Multi-Step Equilibration of Membrane Systems

Following energy minimization, each membrane system was equilibrated using the standard CHARMM-GUI MARTINI membrane equilibration protocol, consisting of multiple MD stages with progressively increasing integration time steps and gradually reduced positional restraints applied to protein atoms and lipid headgroups. This procedure allows gradual relaxation of the lipid bilayer while maintaining structural stability of the protein.

Temperature was maintained at 303.15 K using the velocity-rescaling thermostat (τₜ = 1.0 ps) with separate coupling groups for protein, membrane, and solvent. Pressure was controlled at 1 bar using the Berendsen barostat with semi-isotropic coupling (compressibility = 3 × 10^−4^ bar^−1^). Electrostatic interactions were treated using the reaction-field scheme (εᵣ = 15) with Coulomb and van der Waals cutoffs of 1.1 nm.

### 5.12. Production Runs of Membrane Systems

Production simulations were run for 1 µs per system under NPT conditions. Temperature was maintained at 303.15 K. The Berendsen barostat was replaced by the Parrinello–Rahman barostat [133] for proper NPT ensemble sampling. All electrostatic and van der Waals parameters remained unchanged from equilibration.

### 5.13. Trajectory Analysis

Trajectory analyses were performed using MDAnalysis [134,135]. For each system, a single production trajectory was analyzed without independent replicas. RMSF was calculated over the entire production trajectory for each system. Lipid–protein contacts were defined using a distance cutoff of 4.5 Å between any atom of a given lipid species and any atom of the protein; contact frequencies were computed as the fraction of trajectory frames in which at least one such contact was detected. Secondary structure was assessed on a representative frame extracted from the final portion of each production trajectory using CHARMM-GUI structure visualization; continuous quantitative monitoring of secondary structure evolution was not performed.

## Data Availability

The protein structure used in this study was obtained from the PDB [PDB ID: 4FEB]. All data are publicly available at https://www.rcsb.org/. No additional experimental data were generated.

## Abbreviations

HD	Huntington’s disease
HTT	huntingtin
mHTT	mutant huntingtin
mHTT-Q70	mHTT with 70 polyglutamine (Q70)
polyQ	polyglutamine
CAG	cytosine–adenine–guanine trinucleotide repeat
MD	molecular dynamics
CG-MD	coarse-grained molecular dynamics
RMSF	root mean square fluctuation
PDB	Protein Data Bank
N17	N-terminal 17–residue domain of huntingtin
OPSG	glycolipids
POPC	1-palmitoyl-2-oleoyl-*sn*-glycero-3-phosphocholine
POPE	1-palmitoyl-2-oleoyl-*sn*-glycero-3-phosphoethanolamine
POPS	1-palmitoyl-2-oleoyl-*sn*-glycero-3-phospho-L-serine
POPI	1-palmitoyl-2-oleoyl-*sn*-glycero-3-phosphoinositol
PSM	palmitoylsphingomyelin
PIP_3_	phosphatidylinositol(3,4,5)-trisphosphate
CHOL	cholesterol
